# Encryption of agonistic motifs for TLR4 into artificial antigens augmented the maturation of antigen-presenting cells

**DOI:** 10.1371/journal.pone.0188934

**Published:** 2017-11-30

**Authors:** Masaki Ito, Kazumi Hayashi, Tamiko Minamisawa, Sadamu Homma, Shigeo Koido, Kiyotaka Shiba

**Affiliations:** 1 Division of Oncology, Research Center for Medical Sciences, The Jikei University School of Medicine, Tokyo, Japan; 2 Division of Protein Engineering, Cancer Institute, Japanese Foundation for Cancer Research, Tokyo, Japan; 3 Division of Gastroenterology and Hepatology, Department of Internal Medicine, The Jikei University School of Medicine, Tokyo, Japan; University of Massachusetts Medical School, UNITED STATES

## Abstract

Adjuvants are indispensable for achieving a sufficient immune response from vaccinations. From a functional viewpoint, adjuvants are classified into two categories: “physical adjuvants” increase the efficacy of antigen presentation by antigen-presenting cells (APC) and “signal adjuvants” induce the maturation of APC. Our previous study has demonstrated that a physical adjuvant can be encrypted into proteinous antigens by creating artificial proteins from combinatorial assemblages of epitope peptides and those peptide sequences having propensities to form certain protein structures (motif programming). However, the artificial antigens still require a signal adjuvant to maturate the APC; for example, co-administration of the Toll-like receptor 4 (TLR4) agonist monophosphoryl lipid A (MPLA) was required to induce an *in vivo* immunoreaction. In this study, we further modified the previous artificial antigens by appending the peptide motifs, which have been reported to have agonistic activity for TLR4, to create “adjuvant-free” antigens. The created antigens with triple TLR4 agonistic motifs in their C-terminus have activated NF-κB signaling pathways through TLR4. These proteins also induced the production of the inflammatory cytokine TNF-α, and the expression of the co-stimulatory molecule CD40 in APC, supporting the maturation of APC *in vitro*. Unexpectedly, these signal adjuvant-encrypted proteins have lost their ability to be physical adjuvants because they did not induce cytotoxic T lymphocytes (CTL) *in vivo*, while the parental proteins induced CTL. These results confirmed that the manifestation of a motif’s function is context-dependent and simple addition does not always work for motif-programing. Further optimization of the molecular context of the TLR4 agonistic motifs in antigens should be required to create adjuvant-free antigens.

## Introduction

The immunomodulatory adjuvant is a critical component of a vaccine because it induces the cellular immune response to antigens, which are generally far less immunogenic. Adjuvants can be sub-classified into "physical adjuvants" and "signal adjuvants" from a functional perspective [1, 2, 3]. Functioning of both the signal and physical adjuvants is required for the adjuvants to induce robust cellular immunity *in vivo*.

The physical adjuvant includes inorganic materials such as mineral oil or aluminum salt and assists both the uptake of antigens by antigen-presenting cells (APC) and the proficient induction of an immune response, which requires a T-cell receptor (TCR) and major histocompatibility complex (MHC)/peptide interaction. Although aluminum salt such as Alum is used as a physical adjuvant in many human vaccines, this adjuvant is limited to stimulating Th2-biased antibody responses, which may be inappropriate for the induction of cellular immunity against cancer [3, 4].

An appropriate maturation of APC is necessary to induce robust cellular immunity. The signal adjuvant functions as the immunomodulator, which activates the maturational status of the APC. Lipid A, the biologically active portion of lipopolysaccharide (LPS), is a signal adjuvant that triggers productive cellular immunity [5]. However, the pharmacologic use of purified lipid A as a signal adjuvant is precluded by its toxicity. Toll-like receptor 4 (TLR4) is a pattern recognition receptor of APC and can recognize structurally different lipid A molecules [6]. The toxicity and adjuvant potency of lipid A are determined by the level of phosphorylation, number of acyl chains, and fatty acids [7, 8]. Monophosphoryl lipid A (MPLA) is a lipid A derivative that lacks the phosphate attached at the 1-position on the reducing-terminal glucosamine moiety. MPLA is less toxic than LPS and has been clinically used as an adjuvant for cancer vaccines [5, 9].

The maturation of APC is regulated by signal transduction pathways activated by TLR agonists through the production of cytokines (TNF-α and IL-6), chemokines and the expression of co-stimulatory molecules (CD80, CD86 and CD40) on APC. TNF-α is a potent proinflammatory cytokine produced by activated macrophages and has pleiotropic effects on immune cell survival, activation, and differentiation [10]. CD40 is a co-stimulatory molecule important for antigen presentation, and is expressed by a wide variety of cells including B cells, macrophages and dendritic cells [11].

Shanmugam A. *et al*. have identified several peptides isolated using phage display combinatorial peptide technology, which functionally mimicked LPS [12]. The RS01 (Gln-Glu-Ile-Asn-Ser-Ser-Tyr) and RS09 (Ala-Pro-Pro-His-Ala-Leu-Ser) peptides activated NF-κB signaling through the TLR4 and induced inflammatory cytokine secretion *in vitro* [12]. RS09 also induced an antibody response when the antigen conjugated to Keyhole Limpet Hemocyanin with RS09 was used to vaccinate mice. However, it has not been reported that these peptides function as signal adjuvants for inducing cellular immunity.

We have previously developed the artificial antigen F37A by the combinatorial assembling of peptide epitopes structural peptide motifs, and have shown that F37A evokes robust cellular immunity [2, 13]. Vaccination with F37A containing MPLA (as the signal adjuvant) induces robust antigen-specific CD8^+^ T-cell immune responses and antitumor effects *in vivo* without being combined with a physical adjuvant such as Freund's mineral oil. This antigen is endocytosed into dendritic cells (professional APC) through the scavenger receptor and can strongly present the antigenic epitopes to T cells. In other words, F37A is an antigen having a physical adjuvant function, but not a signal adjuvant function [2]. Therefore, we created an antigen that included the TLR4 agonistic peptide motifs (RS01 and RS09) to impart signal adjuvant function in the antigen itself. We examined whether these antigens could induce the maturation of APC and the antigen-specific CTL response without using any conventional adjuvants. A better understanding of the function of artificial antigens with TLR4 agonistic peptide motifs in the maturation of APC could lead to promising new developments in the design of more effective and rational artificial antigen vaccines.

## Materials and methods

### Cell lines and animals

The RAW264.7 (RAW) and E.G7-OVA (OVA-expressing EL4) cell lines were purchased from American Type Culture Collection and the HEK-Blue human TLR4 cell line (HEK-Blue) was purchased from InvivoGen. The RF33.70 and DC2.4 cell lines were kindly provided by K. L. Rock (University of Massachusetts Medical School, USA). Female C57BL/6 mice were purchased from The Sankyo Labo Service.

### Production of recombinant proteins

**Fig 1:**
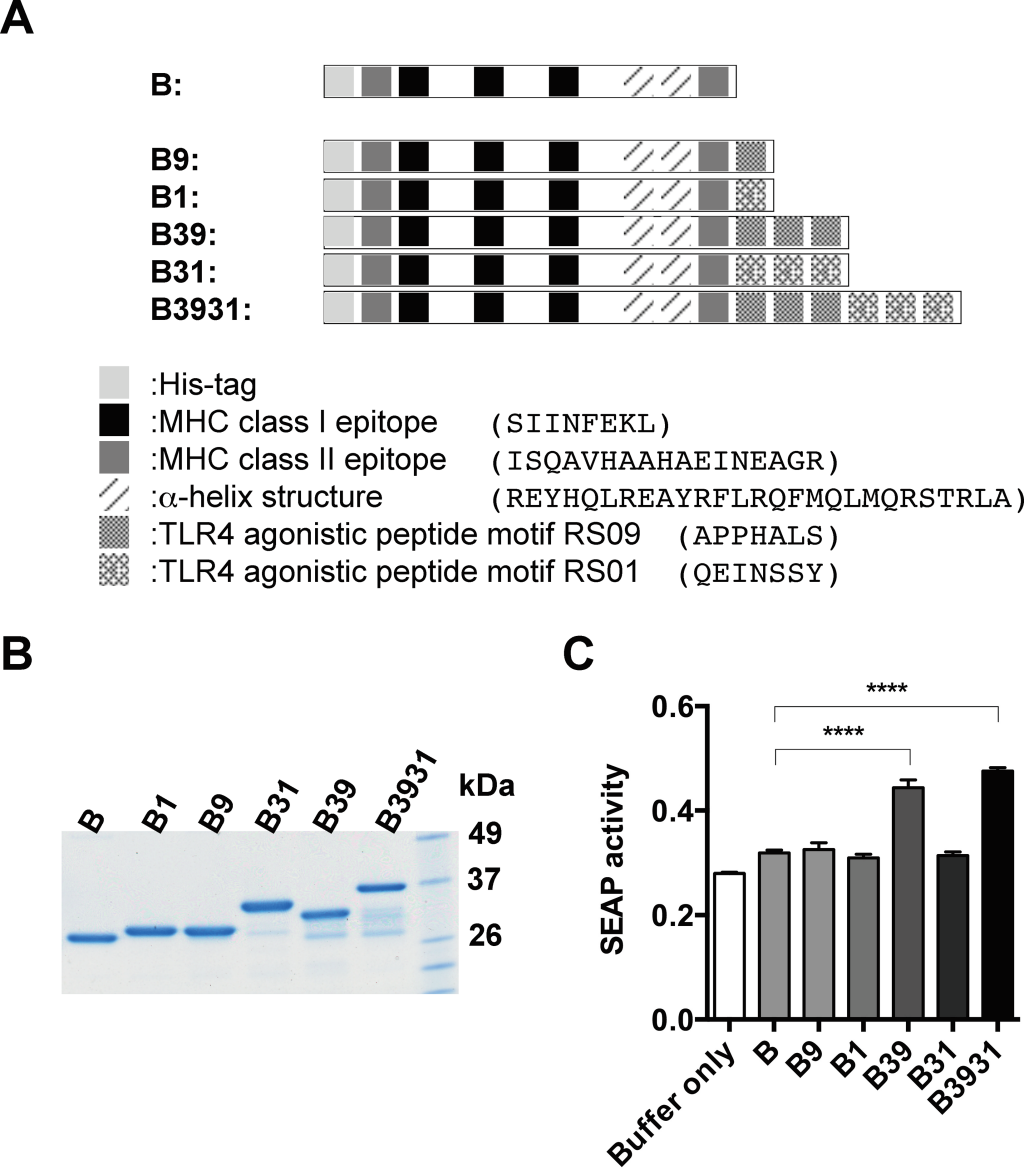
Schematic representation of artificial antigens and NF-κB activation in HEK-Blue cells. (A) Schematic representation of artificial antigens comprising the TLR4 agonistic motifs. We have prepared the antigens by extending the TLR4 agonistic motifs to the C-terminus of antigen B so that they have physical adjuvant function. Antigen B is composed of a 6x His-epitope tag, two MHC class II epitopes, three MHC class I epitopes and α-helix structure motifs. The amino acid sequences of the antigens are shown in S1 Table. (B) Purity of the antigens is shown by a Coomassie blue-stained SDS-PAGE gel. (C) Antigens B39 and B3931 with triple motifs of RS09 at the C-terminus of antigen B, enhanced NF-κB signaling through TLR4 stimulation in HEK-Blue cells. NF-κB activation was determined by SEAP activity. (****, *P* < 0.001 compared with antigen B treatment.).

We selected two types of peptide motifs RS01 (QEINSSY) and RS09 (APPHALS), which are considered to be TLR4 stimulating and hence activators of NF-κB signaling [12], and tethered them to the C-terminus of antigen B (Fig 1A). The genes coding single-, tri- and hexa-peptide motifs were chemically synthesized and subsequently cloned into the modified pKS601 vector with an N-terminal His-tag [2]. Amino acid sequences of the antigens are described in the Supporting Information section, S1 Table. Expression plasmids were transfected the *E*. *coli* ClearColi BL21 strain (Lucigen) which lacks LPS activity. His-tagged proteins were purified by Talon metal affinity resin (Clontech). Protein purity was confirmed by SDS-PAGE. Endotoxin levels were measured with a limulus amebocyte lysate (LAL) assay using an Endosafe-PTS system (Charles River).

### Evaluation of TLR4 agonistic activity using HEK-Blue cells

HEK-Blue cells were used to determine whether recombinant proteins could signal through TLR4. This cell line is derived from HEK293 cells and is carrying human TLR4/MD2/CD14 genes and a SEAP (secreted embryonic alkaline phosphatase gene) reporter construct inducible by NF-κB signaling. Cells were maintained in DMEM with 10% FBS, 50 μg/mL penicillin-streptomycin, 100 μg/mL normocin, and 2 mM L-glutamine supplemented with 1X HEK-Blue Selection reagent (Invivogen). 1.25x10^5^ HEK-Blue cells were plated on 96-well plates in 200 μL final volume and treated with various antigens at 40 μg/mL. After 24 hours of stimulation, NF-κB activation was determined using the detection medium QUANTI-Blue (Invivogen). Briefly, 180 μL of QUANTI-Blue was added to a 96-well flat bottom plate together with 20 μL of culture supernatant from the antigen treated samples. After 2 hours of incubation at 37°C, the SEAP levels were determined using spectrophotometry at 650 nm.

### Immunoblot analysis

RAW cells were incubated with 40 μg/mL antigens or 1 μg/mL LPS (Sigma-Aldrich L2880) for 2 hours. Cells were washed twice with cold PBS and lysed using RIPA buffer with proteinase inhibitor. Total cell extracts were resolved using 4–12% SDS-PAGE gels and analyzed by Western blot using an anti-NF-κB antibody (Cell Signaling Technology, clone 93H1 and clone D14E12).

### Cytokine analysis

RAW cells were incubated with 40 μg/mL antigens or 1 μg/mL LPS for 4 or 20 hours. Cell culture supernatants were assayed for cytokines by ELISA (BioLegend) according to the manufacturer’s standard protocols.

### Flow cytometry analysis

RAW cells were treated with 40 μg/mL antigens or 10 μg/mL LPS for 20 hours, after which they were stained and analyzed by flow cytometry for the expression of the maturation marker CD40 using an anti-CD40 antibody (Serotech, clone 3/23). Fluorescence was analyzed using a MACSQuant analyzer.

### *In vitro* antigen presentation assay

The *in vitro* antigen presentation assay was performed as described previously [2]. In brief, DC2.4 cells (mouse dendritic cell line) were treated with antigens. Then the DC2.4 cells were co-cultured with RF33.70 OVA-specific T-T hybridoma cells (1x10^5^ cells/well). The response of the stimulated RF33.70 cells was assessed based on the amount of IL-2 released into a 100 μL aliquot of culture medium, which was measured using a murine IL-2 ELISA kit (BD Biosciences).

### *In vivo* immunization and tetramer assay

Three mice per group were immunized with antigen B and B3931. Each mouse was injected into the peritoneal cavity (intraperitoneal injection, IP), the dermis of the hind footpad (intradermal injection, ID) and the tail base (subcutaenous injection, SC) three times with 100 μg of antigen with or without MPLA (10 μg per mouse, Sigma-Aldrich L6895) at 14-day intervals. At 7 days after final immunization, all mice were anaesthetized by intraperitoneal injection of sodium pentobarbital solution (50 mg/kg), then euthanized by cervical dislocation for the tetramer assay.

The tetramer assay. Splenocytes (3x10^6^) prepared from immunized mice were incubated for 5 days with 1x10^6^ irradiated (100 Gy) E.G7-OVA cells in 1 ml of RPMI 1640 medium supplemented with 10% FBS and IL-2 (20 ng/mL) in a 24-well culture plate. The frequency of OVA-specific CD8^+^ effector T-cells was determined by tetramer staining *ex vivo*. *In vitro*-stimulated splenocytes were stained with T-select H-2Kb OVA Tetramer-SIINFEKL-PE (MBL, Japan) for 20 min at 4°C according to the manufacturer’s protocol. Fluorescence was analyzed using a MACSQuant analyzer (Miltenyi Biotech).

### Ethics statement

All animal experiments were performed by following a protocol that was reviewed and approved by the Institutional Animal Care and Use Committee at Jikei University (protocol #22-010C3, #22-038C3, #25–052). Briefly, four mice were housed per cage under conventional conditions. Animal rooms were maintained at 22 (± 2)°C and a relative humidity of 55 (± 10) %. Animals remained on 12:12-hour full-spectrum light:dark cycles and received ad libitum food (Rodent Diet CE-2, CLEA Japan) and water. Mice were monitored every 2–3 days. From the standpoint of animal welfare, mice were anaesthetized lightly by intraperitoneal injection of sodium pentobarbital solution (50 mg/kg) and then euthanized by cervical dislocation. If the mouse appeared moribund indicating low probability of surviving for greater than 24 hours, it was also euthanized. In this study, no mice reached the limit of humane endpoint established in our approved animal protocol (20% weight loss).

### Statistical analysis

Data handling, analyses, and graphic representations were performed using Prism 6 (GraphPad). Data summarized in bar diagrams are expressed as mean ± 1 SD and asterisks indicate statistical differences calculated by one-way ANOVA with Dunnett’s multiple comparison test.

## Results

### Production of antigen with TLR4 agonistic peptide motifs

We previously created the artificial antigen F37A that can evoke ovalbumin (OVA)-specific cellular immune responses without an inorganic physical adjuvant such as complete Freund's adjuvant (CFA) [2]. F37A contains antigenic epitope sequences of OVA, OVA-I (class I epitope, SIINFEKL) and OVA-II (class II epitope, ISQAVHAAHAEINEAGR), and the α-helix structure motif which are shown in Fig 1A and S1 Table. In this study we used antigen B, which is different from antigen F37A by only a few amino acid sequences that introduced several restriction sites into F37A for inserting the TLR4 agonistic motifs.

Based on Shanmugam's previous reports that demonstrated potent TLR4 agonistic activity of short peptides we chose two candidates, a RS01 motif (QEINSSY) and a RS09 motif (APPHALS), as signal adjuvants to add the immunostimulatory activity into antigen B [12]. We designed the artificial antigens containing the TLR4 agonistic single-, triple- and hexa-peptide motifs of RS01 or RS09 at the C-terminus of antigen B (Fig 1A and S1 Table). The antigens were produced from the ClearColi endotoxin-free bacterial expression system. The endotoxin levels of prepared antigens were verified as being under 0.5 EU/μg using a LAL assay (data not shown). Purity of the recombinant proteins is shown by SDS-PAGE in Fig 1B.

### TLR4 agonistic peptide motifs in the antigen can impart TLR4 agonist activity in HEK-Blue cells

To determine whether antigens could signal through TLR4, we first examined the activation of TLR4 signaling using HEK-Blue cells. This cell line expresses human TLR4 and contains the secreted embryonic alkaline phosphatase (SEAP) reporter gene which is induced by NF-κB and activator protein-1 (AP-1). The HEK-Blue cells were incubated with purified antigens and then the TLR4-dependent NF-κB activation was measured by SEAP reporter activity (Fig 1C). Among the six antigens tested in HEK-Blue cells, only two antigens (B39 and B3931) showed the NF-κB-SEAP reporter gene activation. Both antigens have tandem tri-RS09 motifs. In contrast, C-terminal extension of proteins with the single RS09 motif (B9), and single (B1) and triple (B31) RS01 motifs did not induce the TLR4 agonist activity (Fig 1C). These results suggested that a single TLR4 agonistic motif in the proteinous antigen is insufficient to stimulate NF-κB activation through the TLR4 in HEK-Blue cells.

### NF-κB activation is stimulated by antigen containing TLR4 agonistic peptide motifs

**Fig 2:**
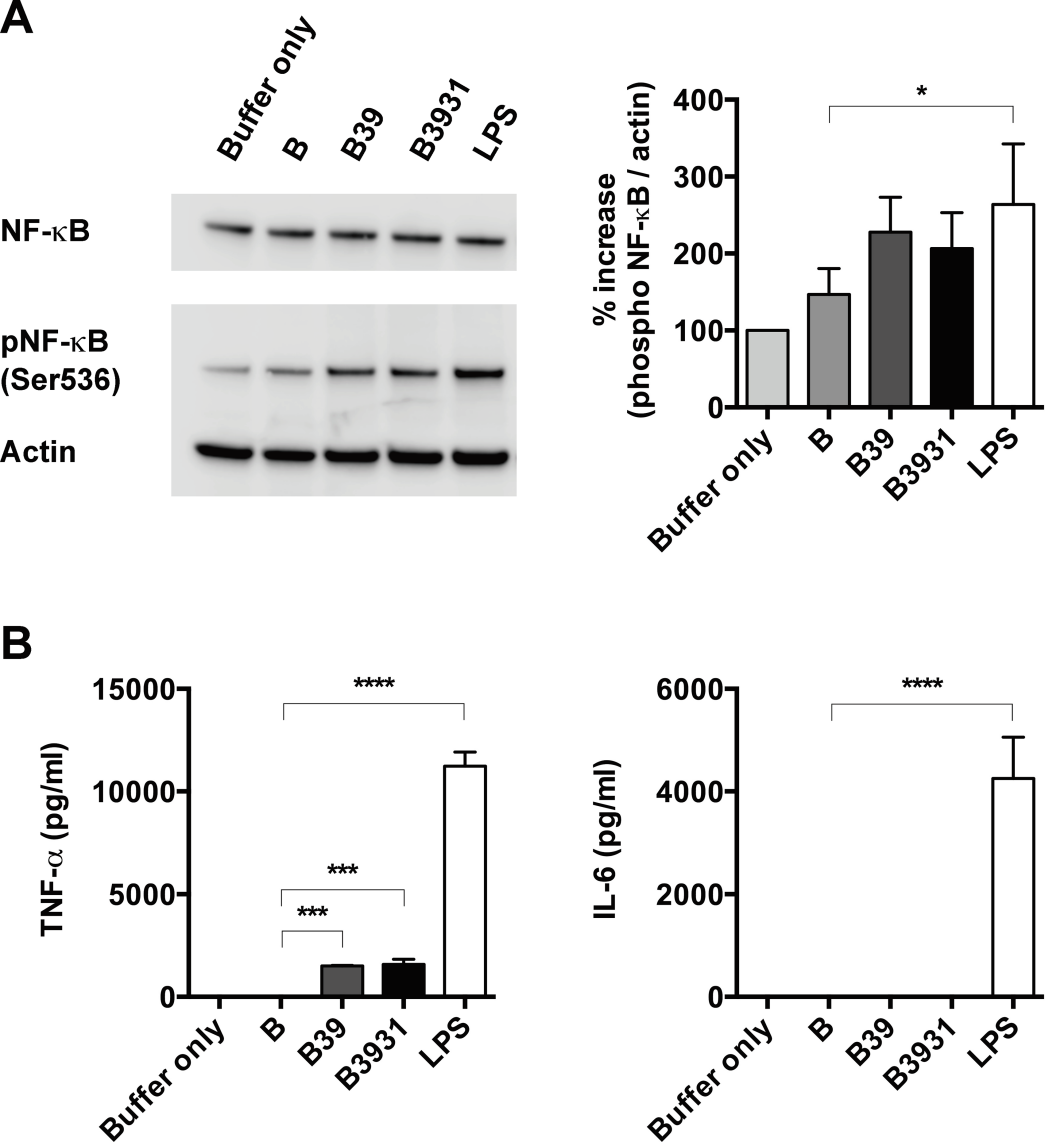
Antigens B39 and B3931 activate the NF-κB signaling pathway and induce TNF-α expression in RAW264.7 macrophage cells. (A) Antigens B39 and B3931 induced a slight phosphorylation of Ser-536 of NF-κB in RAW264.7 macrophage cells. (*, *P* < 0.05 compared with antigen B treatment.) The levels of phospho-NF-κB (S536) and β-actin were analyzed by immunoblotting. A representative Western blot from three independent experiments is shown (left panel). The intensity of the bands corresponding to phospho-NF-κB and β-actin was quantified by densitometry (right panel). (*, *P* < 0.05 compared with antigen B treatment.) (B) TNF-α expression, but not IL-6 expression, was induced in RAW cells by treatment with antigens B39 and B3931 for 4 hours. Reactions were performed in triplicate. (***, *P* < 0.005; ****, *P* < 0.001 compared with antigen B treatment.).

NF-κB phosphorylation is a hallmark of the inflammatory response controlled by the TLR4 signaling pathway. Therefore, we verified the phosphorylation levels of NF-κB p65 in a murine macrophage cell line (RAW cells) to determine the extent of NF-κB activation. The level of phosphorylation of NF-κB p65 (Ser536) was significantly increased by stimulation with LPS (Fig 2A). Phosphorylation of NF-κB was also elevated in response to antigens B39 and B3931. The statistical significance between B and LPS was P < 0.05, whereas between B and B39 or B and B3931 it was P > 0.05. However, taken together with the results of the NF-κB-SEAP reporter gene activation by antigens B39 and B3931 in HEK-Blue cells, we conclude that antigens B39 and B3931 that contain the triple-TLR4 agonistic RS09 motif at the C-terminus do activate NF-κB signaling pathways in RAW cells, even without stimulation with lipid A or LPS.

### TNF-α expression is induced by artificial antigens

TNF-α is an important inflammatory cytokine that has been implicated in APC maturation. We also examined cytokines (TNF-α and IL-6) released into the cell culture medium of RAW cells. The induction of TNF-α occurred as early as 4 hours after treatment with antigens B39 and B3931 (Fig 2B). Significant levels of IL-6 were observed for LPS treatments at 4 and 20 hours, but not for antigens B39 and B3931 (Fig 2B and S1A Fig). Chemokine production of RNATES (CCL5) and MDC (CCL22) was also increased in RAW cells treated by antigens B39 and B3931 for 20 hours (S1B Fig). These results suggested that the B39 and B3931 antigens activated the TLR4-NF-κB signaling pathway and function as the immunostimulatory mediators for producing inflammatory cytokines. Furthermore, the lack of IL-6 expression suggested that B39 and B3931 might be induced the cytokine production through the signaling pathways different from LPS in RAW cells.

### CD40 expression on RAW cells is promoted by artificial antigens

**Fig 3:**
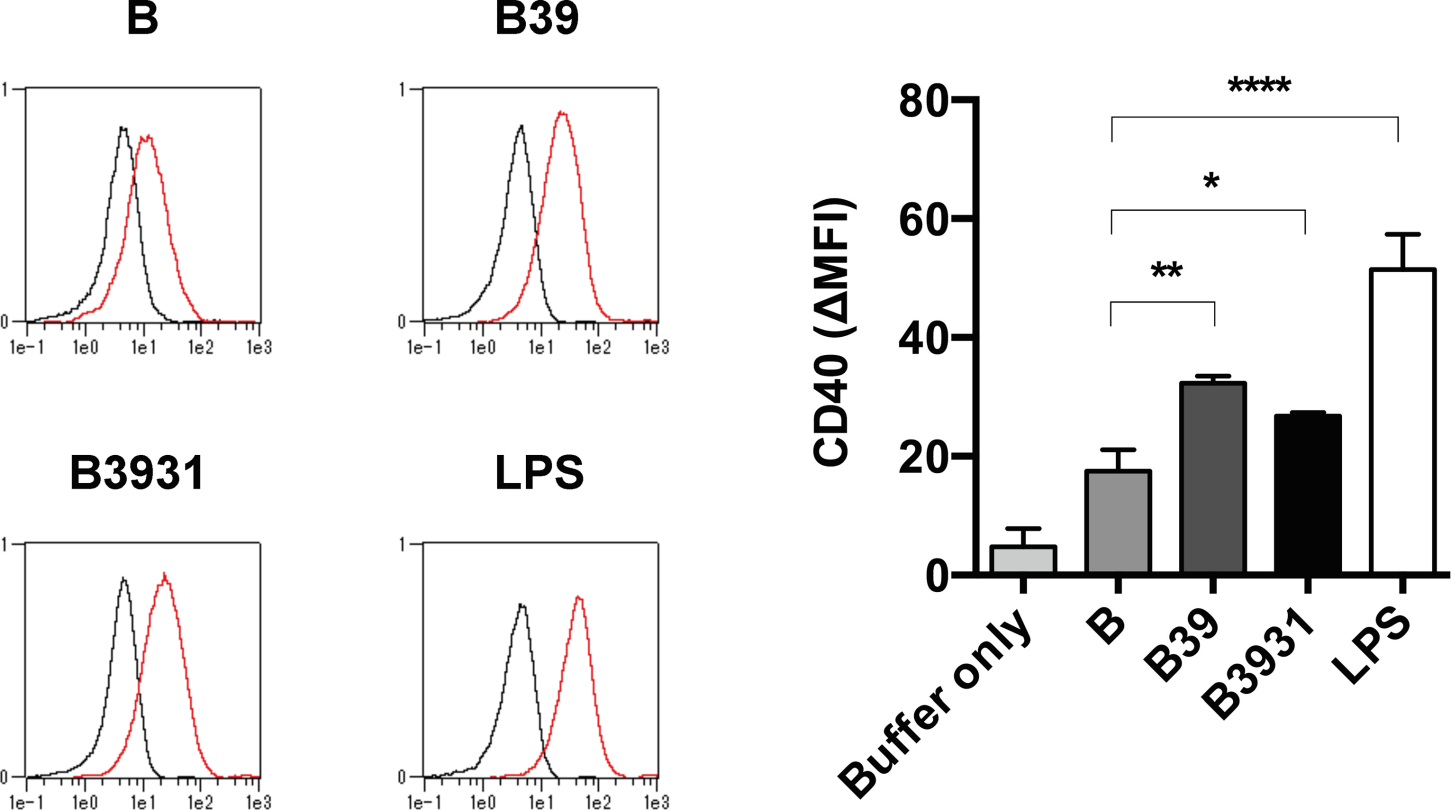
CD40 expression was up-regulated with the artificial antigens in RAW cells. Representative flow cytometry histograms from three independent experiments showing the level of expression of the maturation marker CD40 (red) and isotype-matched control antibody (black) (left panel). The bar graph represented the mean and standard deviation of ΔMFI calculated by subtracting the isotype control MFI from the CD40 MFI (right panel). (*, *P* < 0.05; **, *P* < 0.01; ****, *P* < 0.001 compared with antigen B treatment.).

Up-regulation of cell surface co-stimulatory molecules CD40, CD80, CD86 and MHC class II are markers of the mature differentiation state of APC. To evaluate the effect of artificial antigens on APC maturation, we measured the expression of these maturation markers on RAW cells. Flow cytometry analysis showed that CD40 expression on RAW cells was significantly up-regulated by the antigens B39 and B3931 with TLR4 agonistic motifs (Fig 3). An increase of CD80, CD86, and MHC class II maturation markers were weakly detected in both B39 and B3931 antigens, with no significant difference in expression (data not shown).

### Signal adjuvant activity of the TLR4 agonistic motif is not enough to induce significantly stronger cellular immunity *in vivo*

**Fig 4:**
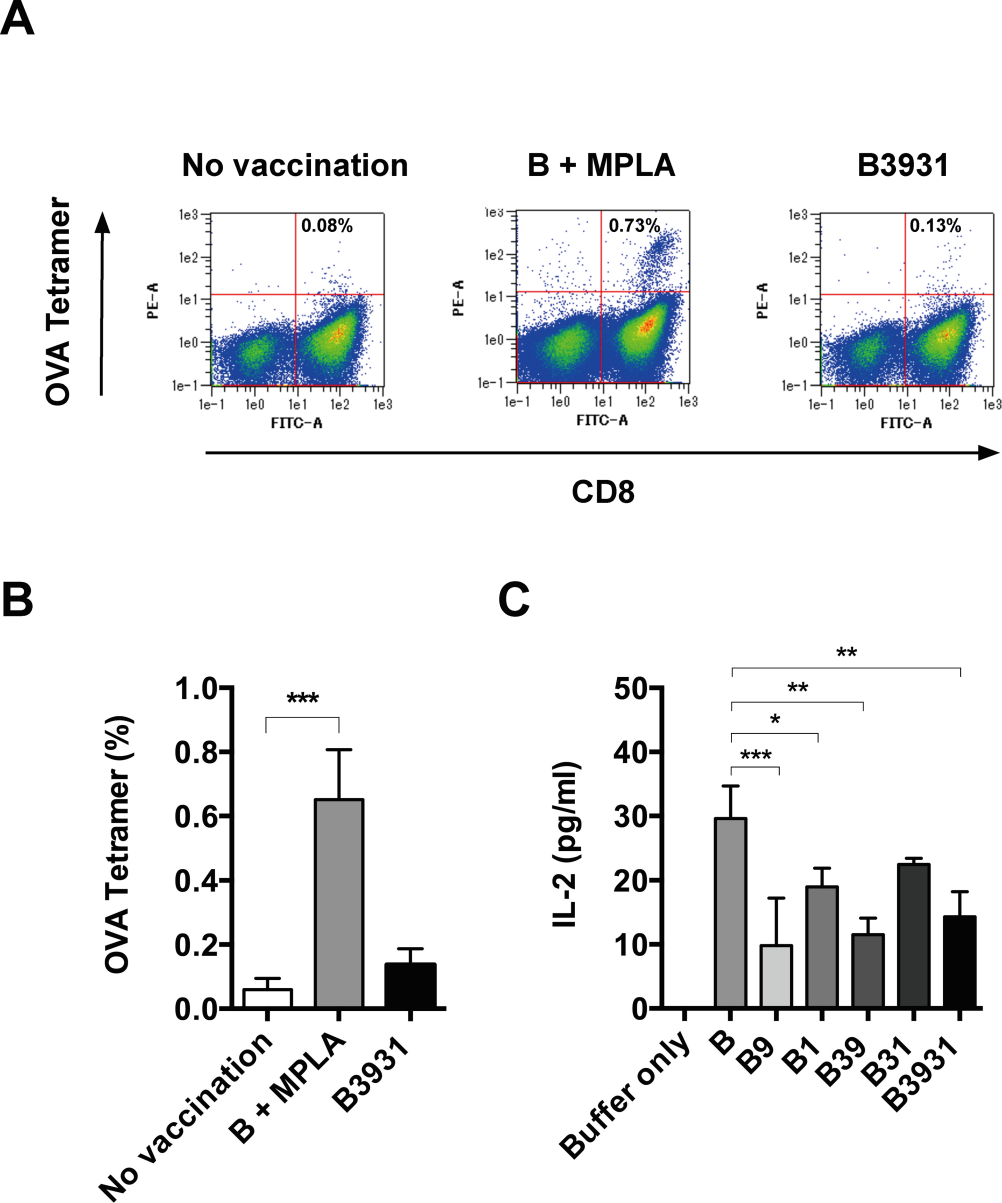
Induction of antigen-specific CD8^+^ T cells *in vivo* and antigen presentation *in vitro*. (A) A representative flow cytometry analysis performed in a tetramer assay. Tetramer positive OVA-specific T cells were not significantly increased in mice immunized with antigen B3931 alone. The frequencies of double positive populations: OVA tetramer (+) and CD8 (+) cells are shown in the upper right frame of the density plot. (B) A significant increase in the OVA-specific (tetramer positive) CD8^+^ T-cells was observed following immunization with B + MPLA, but not observed with immunization of antigen B3931 alone via IP. Bars represent mean ± SD for a group of 3 mice. (***P<0.005 compared with no vaccination.) (C) Antigen presentation of B39 and B3931 was reduced compared to antigen B *in vitro*. Antigen presentation activity was determined by measuring the level of IL-2 produced from the RF33.70 cells. Reactions were performed in triplicate. (*, *P*<0.05; **, *P*<0.01; ***, *P*<0.005 compared with antigen B treatment.).

To evaluate the ability of artificial antigens to induce cellular immunity *in vivo* without conventional adjuvants, we investigated whether antigen B3931 induced the antigen-specific CD8^+^ T cells *in vivo*. As previously reported, vaccination with antigen B plus MPLA provided a strong induction of OVA-specific tetramer positive-CD8^+^ T cells in mice (Fig 4A and 4B) [2]. Conversely, the vaccination of antigen B3931 alone did not induce a significantly strong antigen-specific immune response *in vivo*. The vaccination route has been shown to have a significant influence on immune responses. The effect of the injection route was evaluated by administering antigen B3931 to mice by IP, ID, and SC routes. However, antigen B3931 did not increase the *in vivo* numbers of antigen-specific CTL, regardless of vaccination route (S2 Fig). We also measured the cytotoxic activity of splenocytes using a standard ^51^Cr releasing assay against OVA-positive thymoma EG7-OVA cells. However, no significant cytotoxicity was observed when the mice were vaccinated with antigen B3931 alone (data not shown).

### Addition of TLR4 agonistic motifs to the C-terminal end of artificial antigens may affect physical adjuvant activity

The vaccination of antigen B3931 alone failed to induce cellular immunity, so we re-checked the physical adjuvant activity of antigens *in vitro*. The antigen-presenting ability was evaluated by IL-2 production from RF33.70 cells (OVA-specific T cells) when the RF33.70 cells and the antigen treated DC2.4 cells (dendritic cells) were co-cultured. The IL-2 production by antigens B39 and B3931 was lower than the original antigen B (Fig 4C). These results suggested that the addition of the TLR4 agonistic RS09 motifs at the C-terminal end of the antigen reduced its endocytotic ability.

## Discussion

TLR4 can recognize lipid A’s diverse structures by the number and length of the fatty acid chains and the presence of terminal phosphate residues [6]. In this study, TLR4 agonistic peptide motifs were applied to the C-terminus of antigen B, in order to mimic the structural pattern of lipid A [12]. Among those, only antigens B39 and B3931, which had tri-RS09 motifs, enhanced the activation of NF-κB in HEK-Blue cells (Fig 1C). The presence of a single-RS09 motif did not induce agonist activity of TLR4. These results suggested that the tandem tri-RS09 motif repeat structures in an antigen may mimic the molecular conformation of lipid A.

TNF-α is a cytokine principally secreted from macrophages and is critical for activating the adaptive immune response. A significant increase in secreted cytokine TNF-α was already observed only 4 hours after exposure to B39 and B3931 in RAW cells (Fig 2B). While LPS is a strong TLR4 ligand and stimulated the rapid and robust production of IL-6 (Fig 2B), antigens B39 and B3931 did not affect IL-6 secretion. The autocrine stimulation by TNF-α could be responsible for the expression of CCL22 and CCL5 after 20 hours of stimulation (S1B Fig). IL-6 is involved in macrophage differentiation and inhibits Th1 polarization [14]. LPS induces strong inflammatory cytokine production through both MyD88- and TRIF-dependent TLR4 signaling. On the other hand, MPLA preferentially induces TLR4 signaling through the TRIF-dependent signaling pathway, resulting in the stimulation of beneficial immune responses without the excessive production of pro-inflammatory cytokines [5]. These results suggested that TNF-α expression by antigens B39 and B3931 might activate through different TLR4 signaling pathways than LPS.

Maturation of the APC requires up-regulation of i) MHC molecules; ii) co-stimulatory molecules, including CD40, CD80, and CD86; and iii) cytokines, including TNF-α. Treatment of RAW cells with antigens B39 or B3931 resulted in up-regulation only of the CD40 co-stimulatory molecule (Fig 3), whereas the levels of CD80, CD86, and MHC class II were unchanged (data not shown). CD40, which is expressed on APC and plays important roles in innate immunity, is known to be induced by LPS or TNF-α [15]. Little is known about the mechanisms underlying the stimulation of CD40 by these molecules [16]. Iijima *et al*. have reported that thiol antioxidants, such as N-acetyl-L-cysteine and reduced glutathione, selectively reduce the expression of the CD40 induced by TNF-α in dendritic cells, but have no appreciable effect on the expression of CD80, CD86, and MHC class II [17]. These authors suggested that a post-transcriptional pathway, such as translation and/or degradation of CD40 protein, regulates the reduction of CD40 expression by reducing agents. Moreover, Mann *et al*. have shown that NF-κB and AP-1 are downstream transcriptional mediators of CD40-induced IL-6 gene expression in dendritic cells [18]. These authors suggested that the short-lived activation of NF-κB and activator protein-1 (AP-1) eventually result in a repression of IL-6 gene transcription.

In the present study, HEK-Blue cells containing the SEAP reporter were induced by both NF-κB and AP-1 transcription factors, and we showed that antigens B39 and B3931 have TLR4 agonist activity. Moreover, the production of IL-6 was not induced in RAW cells by the treatment with antigens B39 and B3931. Taken together, our data and the previous study by Mann *et al*. indicated that the differential up-regulation of CD40 in RAW cells by antigens B39 and B3931 might be partially dependent on the signal intensity of the NF-κB and JNK/AP-1 pathway. However, further studies are needed to understand the molecular mechanisms by which antigens B39 and B3931 enhance the differential expression of CD40.

Interestingly, in spite of antigen B not showing SEAP activity in HEK-Blue cells (Fig 1C) and rapid TNF-α induction in RAW cells (Fig 2B), antigen B stimulated CD40 expression (Fig 3). Antigen B was created by embedding various peptide motifs within protein sequences using our motif-programming technology [2, 13]. Although the precise mechanism is not presently known, the motif's molecular context of antigen B may be involved in the activation of the signaling pathways leading to CD40 expression.

On the basis of our *in vitro* studies, antigens B39 and B3931 retain their signal adjuvant function in the maturation of APC. However, we could not detect the ability of the B3931 antigen to stimulate CTL expansion *in vivo* (Fig 4B). Therefore, we re-examined the physical adjuvant function of antigens. As a result, antigens B9, B39 and B3931 having the RS09 motifs, showed a reduction of antigen presenting ability (Fig 4C). It is possible that the addition of the RS09 motifs to the antigen's C-terminal affect the physical adjuvant function of antigen B. Our previous study demonstrated that the physical adjuvant function of antigen B was invoked via the enhancement of antigen uptake through the type A scavenger receptor (SR-A) [2]. It is known that SR-A is involved in the LPS-induced inflammatory response through the TLR4-mediated NF-κB activation pathway. Both SR-A and TLR4 are expressed on the cell surface of APC including macrophages, and SR-A may act as a co-receptor for TLRs [19]. Therefore, the binding of TLR4 agonistic motifs by antigens to the TLR4 may lead to a reduction in the uptake of antigens through the SR-A. The insufficient physical adjuvant activity of antigen B3931 is thought to be one reason why B3931 was not able to elicit cellular immunity *in vivo*.

Antigen B was previously created by our motif-programming technology by the combinatorial combining of antigenic epitope motifs with α-helical structural motifs, to incorporate the novel physical adjuvant function into the antigen itself [2, 13]. In this study, we have synthesized a series of antigens in which TLR4 agonistic RS09 motifs were fused onto the C-terminus of antigen B. We would be able to obtain antigens with the properties of both physical and signal adjuvants by re-using this motif-programming technology without using the scheme of C-terminal extension of antigen B with the RS09 motifs.

## Conclusions

We showed that artificial antigens with TLR4 agonistic motifs could modulate the maturation of APC represented by the expression of inflammatory cytokine TNF-α and the surface maturation marker CD40. However, the signal adjuvant activity and the level of activation of APC maturation with these antigens was much lower than that obtained with the classical strong TLR4 agonist LPS. It is necessary to optimize the physical and signal adjuvant functions for the rational design and synthesis of artificial antigens containing the immunomodulating adjuvant function.

## Supporting information

S1 Fig Cytokine and chemokine production in RAW cells. RAW cells were incubated with 40 μg/mL artificial antigens or 1 μg/mL LPS for 20 hours. (A) TNF-α expression, but not IL-6 expression, was induced by the treatment of antigens B39 and B3931 for 20 hours. (B) Cell culture supernatant was assayed for cytokines and chemokines using the Mouse TLR-induced Cytokines II: Microbial-induced Multi-Analyte ELISArray Kit (Qiagen) according to the manufacturer’s standard protocols.
(TIFF)

S2 Fig Induction of antigen-specific CD8^+^ T cells *in vivo*. (A) A representative flow cytometry analysis performed in a tetramer assay. The frequencies of double positive populations: OVA tetramer^+^ and CD8^+^ cells showed in the upper right frame of the density plot. Tetramer positive OVA-specific T cells were not significantly increased in mice immunized with antigen B3931 alone. B3931 was injected into the peritoneal cavity (IP), the dermis of the hind footpad (ID) and the tail base (SC). (B) A significant increase in the OVA-specific (tetramer positive) CD8^+^ T-cells was observed following immunization with B + MPLA (IP), but not observed with immunization of antigen B3931 (IP), B3931 (ID) and B3931 (SC). Bars represent mean ± SD for a group of 4 mice. (*P<0.05 compared with no vaccination.) Data was analyzed using the non parametric Kruskal-Wallis test.
(TIFF)

S1 Table Amino acid sequences of artificial antigens used in this study. Amino acid sequences are shown by the one-letter code for the amino acid.
(TIFF)
