# Supplementary figures and images for: Encryption of agonistic motifs for TLR4 into artificial antigens augmented the maturation of antigen-presenting cells
Source: PLoS One. 2017 Nov 30;12(11):e0188934. doi: 10.1371/journal.pone.0188934 (PMC5708714; doi:10.1371/journal.pone.0188934)

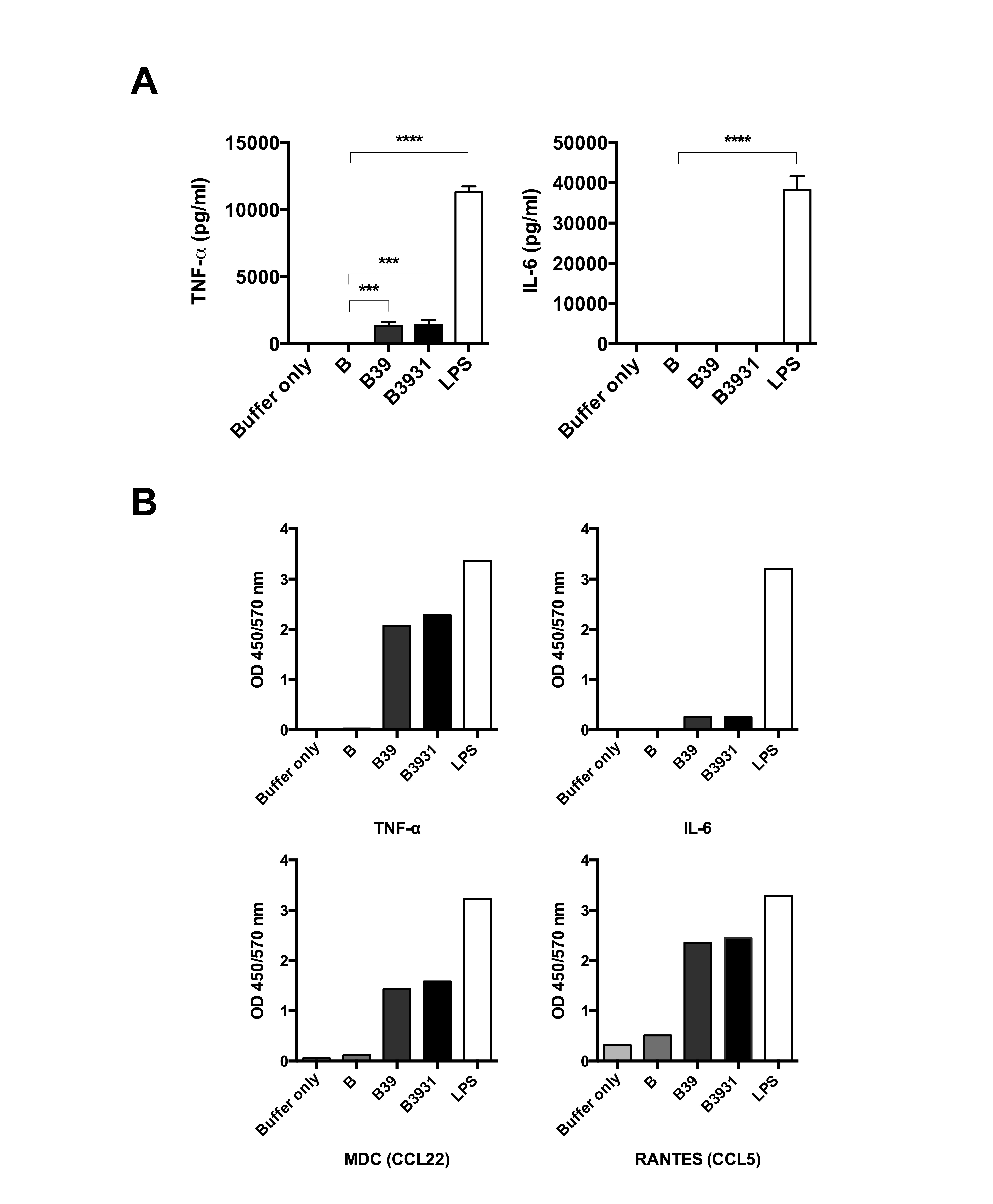

Supplement: S1 Fig — RAW cells were incubated with 40 μg/mL artificial antigens or 1 μg/mL LPS for 20 hours. (A) TNF-α expression, but not IL-6 expression, was induced by the treatment of antigens B39 and B3931 for 20 hours. (B) Cell culture supernatant was assayed for cytokines and chemokines using the Mouse TLR-induced Cytokines II: Microbial-induced Multi-Analyte ELISArray Kit (Qiagen) according to the manufacturer’s standard protocols. (TIFF) [file pone.0188934.s001.tiff]

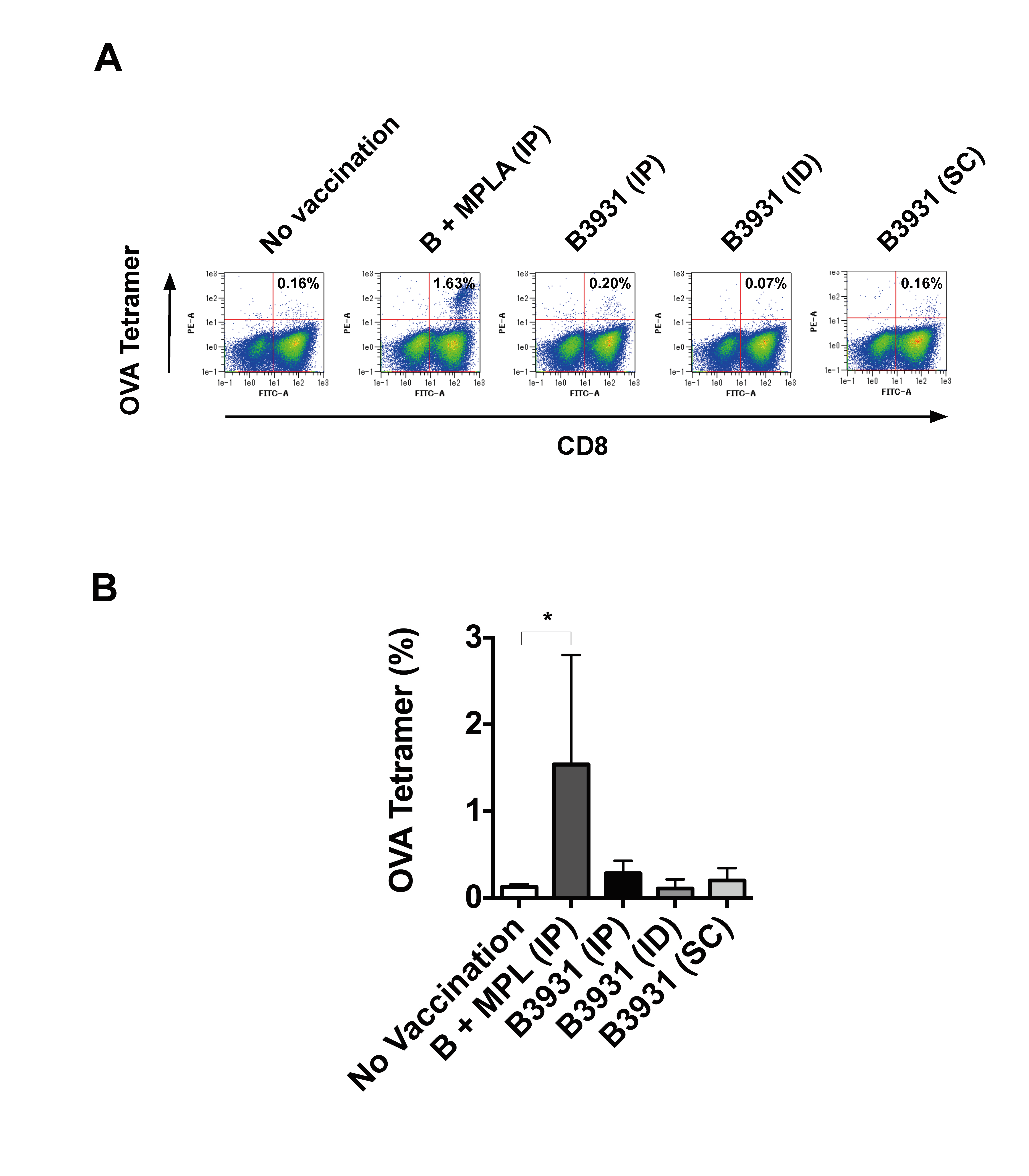

Supplement: S2 Fig — (A) A representative flow cytometry analysis performed in a tetramer assay. The frequencies of double positive populations: OVA tetramer+ and CD8+ cells showed in the upper right frame of the density plot. Tetramer positive OVA-specific T cells were not significantly increased in mice immunized with antigen B3931 alone. B3931 was injected into the peritoneal cavity (IP), the dermis of the hind footpad (ID) and the tail base (SC). (B) A significant increase in the OVA-specific (tetramer positive) CD8+ T-cells was observed following immunization with B + MPLA (IP), but not observed with immunization of antigen B3931 (IP), B3931 (ID) and B3931 (SC). Bars represent mean ± SD for a group of 4 mice. (*P<0.05 compared with no vaccination.) Data was analyzed using the non parametric Kruskal-Wallis test. (TIFF) [file pone.0188934.s002.tiff]

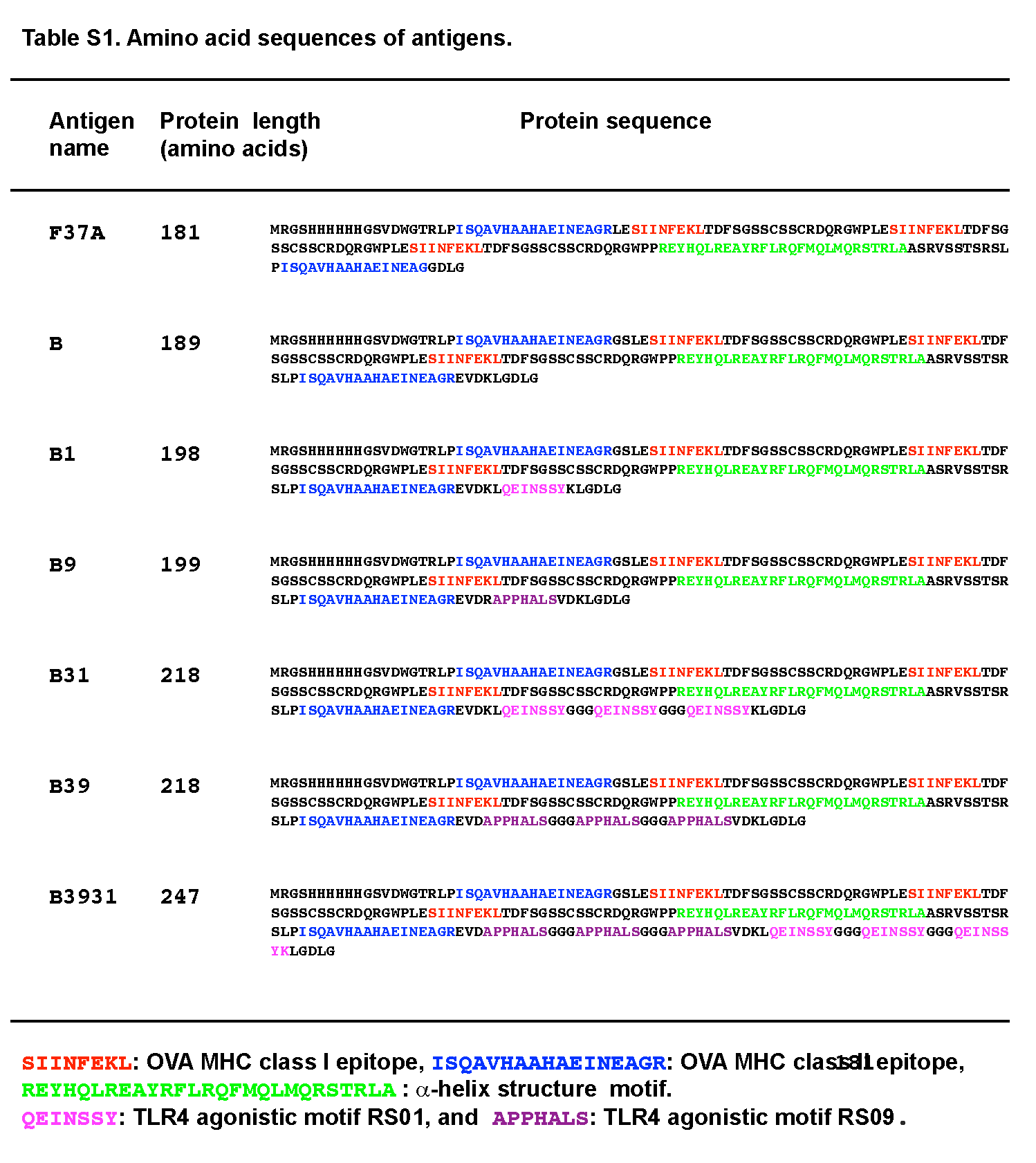

Supplement: S1 Table — Amino acid sequences are shown by the one-letter code for the amino acid. (TIFF) [file pone.0188934.s003.tiff]
